# Incidence and predictors of tuberculosis among HIV patients after initiation of antiretroviral treatment in Ethiopia: a systematic review and meta-analysis

**DOI:** 10.1186/s41182-021-00306-2

**Published:** 2021-02-25

**Authors:** Melkalem Mamuye Azanaw, Nebiyu Mekonnen Derseh, Gebeyehu Shumuye Yetemegn, Dessie Abebaw Angaw

**Affiliations:** 1 Department of Social and Public Health, College of Medicine and Health Sciences, Debre Tabor University, Debre Tabor, Ethiopia; 2 University of Gondar Comprehensive Specialized Hospital, Gondar, Ethiopia; 3 Department of Epidemiology and Biostatistics, Institute of Public Health, College of Medicine and Health Science, University of Gondar, Gondar, Ethiopia

**Keywords:** Incidence, TB, HIV, After ART initiation and Ethiopia

## Abstract

**Background:**

Tuberculosis is the oldest infectious disease and it is still the leading cause of morbidity and mortality worldwide. Even though several primary studies have been conducted on the incidence of tuberculosis among HIV-infected individuals in Ethiopia, national-level tuberculosis incidence is unknown. Therefore, this study is aimed to assess the TB incidence rate and its predictors among HIV-infected individuals after the initiation of ART in Ethiopia.

**Methods:**

We conducted an extensive search of literature as indicated in the guideline of reporting systematic review and meta-analysis (PRISMA). The databases used were PubMed, Google Scholar, and HINARI literature. We used the Joanna Briggs Institute (JBI) Meta-Analysis of Statistics Assessment and Review Instrument for critical appraisal of studies. The meta-analysis and Meta regressions were conducted using STATA 14 software. Met-analysis and meta-regression were computed to present the pooled incidence rate and predictors of tuberculosis among HIV-infected patients after initiation of ART with a 95% confidence interval.

**Results:**

Among a total of 189 studies, 11 studies were included in this analysis. The estimated pooled incidence rate of TB per 100-person year observation (PYO) among HIV-infected patients after initiation of ART therapy was 4.8(95% CI 3.69–5.83). In subgroup analysis, the estimated pooled incidence of tuberculosis showed a slight difference between adults and children after initiation of ART treatment, which was 4.3 (95% CI 2.96, 5.71) and 5.0 (95% CI 3.51, 6.50), respectively. Significantly pooled estimates of predictors of TB incidence by a meta-analysis were being anemic (2.30, 95% CI 1.75, 3.02); on clinical stages III and IV (2.26, 95% CI 1.70, 3.02); and not on cotrimoxazole preventive therapy (CPT) (2.16, 95% CI 1.23, 3.72). Besides, a meta-regression revealed that CD4 <200 cells/mm^3^ (2.12, 95% CI 1.17, 3.86) was a positive significant predictor of TB among HIV patients after the initiation of ART.

**Conclusions:**

The current study showed that the pooled incidence of TB among HIV patients was found to be lower than the WHO 2018 national estimate. Being anemic, WHO stages III and IV, not on CPT, CD4<200cells/μl, and being male were significant predictors of tuberculosis. Therefore, the existing strategies to decrease TB should be strengthening.

**Study protocol registration:**

CRD42020155573.

**Supplementary Information:**

The online version contains supplementary material available at 10.1186/s41182-021-00306-2.

## Background

Tuberculosis (TB) is the oldest infectious disease and it is still the leading cause of morbidity and mortality worldwide [1]. Human immunodeficiency virus-infected people are at the highest risk for developing TB of which 22 times more likely to be ill with TB than those without HIV. Human immunodeficiency virus infection is the main risk factor for active tuberculosis disease [2] and 10% higher rates of progression from latent to active TB that leads to more severe disseminated disease presentation and increased mortality. An estimated 862, 000 people with HIV fell ill TB in 2018 and 251,000 deaths [3].

Despite the widespread use of ART with isoniazid preventive therapy (IPT), intensified case finding, and infection control as a whole the three WHOI’s strategy in Ethiopia, tuberculosis is the common cause of morbidity and mortality among HIV patients. According to the WHO global TB report, 2018, Ethiopia had an estimated 12000 incident TB per 100,000 population among HIV infected individuals [1].

Although HIV itself is the main risk factor for active TB disease, there are possible additional aggravating factors. Based on previous studies, low CD4 count, advanced WHO clinical stage, ambulatory or bedridden functional status, being anemic, undernutrition, OIs, DM, high viral load, and increased family size in adults were positive predictors of TB incidence, and the above plus delayed motor development and inappropriate vaccination for BCG were the predictors of incident TB cases among individuals on ART [4–9], while both cotrimoxazole preventive therapy (CPT) and IPT reduced TB incidence [7, 10–15].

Different efforts have been implemented to integrate TB diagnosis and treatment with HIV care to manage TB among HIV-infected individuals, though TB still occur in HIV patients who are on antiretroviral treatment. Although several primary studies have been conducted on the incidence of TB among HIV-infected individuals in Ethiopia, there are no aggregated national-level studies after ART initiation. Therefore, this systematic review and meta-analysis aimed to estimate the pooled incidence and predictors of tuberculosis among HIV-infected individuals after ART initiation in Ethiopia so as important to design interventions and prevention mechanisms for further improvement of quality of life among people living human immunodeficiency virus (PLHIV). Furthermore, this study will inform health workers and other stakeholders to make early screening and detection of TB in PLHIV as TB can occur in any course of HIV treatment.

## Methods

### Reporting and protocol registration

This systematic review and meta-analysis was reported as indicated in the guideline of reporting systematic review and meta-analysis (PRISMA) [16]. The protocol used in this study was registered with the International Prospective Register of Systematic Reviews (PROSPERO) and can be accessed with the registration number CRD42020155573.

### Searching strategy

We used search engines PubMed, HINARI, Google scholar, and free Google databases in parallel using search strings adapted to the requirements of each database. We conducted search for PubMed/Medline search engine by using the entry term as: (((((“knowledge”[MeSH Terms] OR “knowledge”[All Fields]) AND (“attitude”[MeSH Terms] OR :attitude”[All Fields])) AND (“perception”[MeSH Terms] OR “perception”[All Fields])) AND “practice”[All Fields]) AND (“severe acute respiratory syndrome coronavirus 2”[Supplementary Concept] OR “severe acute respiratory syndrome coronavirus 2”[All Fields] OR “ncov”[All Fields] OR “2019-nCoV”[All Fields] OR “COVID-19”[All Fields] OR “SARS-CoV-2”[All Fields] OR ((coronavirus[All Fields] OR “cov”[All Fields]) AND 2019/11[PubDate] AND (“students”[MeSH Terms] OR “students”[All Fields]). We used specific-subject headings for the other databases (HINARI and Google Scholar). Besides, to identify other relevant articles, we manually searched the reference lists of eligible articles.

### Eligibility criteria

Three independent reviewers (MM, NM, and GS) screened all titles and abstracts of the articles before the full-text review. Full texts were then screened by the same reviewers according to the pre-specified inclusion and exclusion criteria. The majority (two thirds) of the reviewers’ agreement has been taken for screened paper and eligibility for systematic review and meta-analysis.

### Inclusion criteria

We included only people who took ART as study population, reporting as TB incidence rate and predictors among HIV patients, articles published in English language, studies done in Ethiopia, and articles published between 1st January 2010 and 31st October 2019.

### Exclusion criteria

We excluded reviewed papers for the same objectives, those papers exclusively reporting multi-drug or extensively drug-resistant (MDR/XDR), TB as outcomes, and studies other than in humans.

### Extraction of data from eligible papers

Data were extracted for each included study as the name of the first author, date of publication, study setting, target population, study region, study area, study design, sample size, diseased (new TB case), person-year observation, incidence rate per 100-person-year observation, and predictors (risk estimate (HR) and their 95% confidence interval) by using standardized data extraction form (Table 1). Data extraction from selected articles was done independently by three reviews. Disagreements were resolved by 2/3 of the reviewers’ consensus.

**Table 1:** Characteristics of included studies with the outcome of the studies (*n* = 11)

The author with a publication year	Study region	Study design	Study population	Follow-up time (months)	Sample size	TB case	Total PYO	IR per 100 PYO	Study quality
Ayalaw et al., 2015 [13]	Amhara	RFS	Children	72	271	52	1100.5	4.73	Good
Endalamaw et al., 2018 [17]	Amhara	RFS	Children	60	352	34	1294.7	2.63	Good
Beshir et al., 2019 [15]	Oromia	RFS	Children	60	428	67	1109.6	6.04	Good
Alemu et al., 2016 [18]	Amhara	RFS	Children	60	645	79	1854.0	4.26	Good
Jerene et al., 2017 [19]	AA&SNNP	RFS	Adults	84	660	64	2843.5	2.25	Good
Ahmed et al., 2017	Afar	RFS	Adults	84	451	119	1377.4	8.64	Good
Temesgen, 2017	Amhara	RFS	Adults	60	492	83	1285.5	6.46	Good
Dalbo and Tamiso, 2016 [20]	SNNP	RFS	Adults	72	496	106	1977.6	5.36	Good
Assefa et al., 2014 [21]	Amhara	RFS	Adults	42	400	136	1181.8	2.20	Good
Alene et al., 2013 [11]	Amhara	RFS	Adults	60	470	26	1724.1	7.89	Good
Kassa et al., 2012 [22]	AA	RFS	Adults	60	4210	270	8792.3	3.07	Good

*RFS* retrospective follow-up study, *PMO* person month observation, *IR* incidence rate

### Quality assessment for studies

Study quality was assessed using a standardized tool adapted from the JBI tool for cohort studies. The tool was considering the following study characteristics: sampling representative and size, exclusion of TB at cohort entry, outcomes ascertainment during follow-up, and duration of follow-up. Studies fulfilling the required criteria as score 1 and studies with scores 0 were considered to be of poor quality for specified criteria. No studies were excluded from the reviews based on their quality scores [23].

### Data management and processing

The outputs from the searching engines were imported into Endnote Version 7.1 software and duplicates were removed. Data were recorded on the abstraction forms and entered into an Excel 2013 sheet (Microsoft Corporation, WA, USA) and then exported to Stata version 14 for analysis.

### Data synthesis and analysis

Both systematic review and meta-analysis were done by using STATA 14 software. In the qualitative part of the review, all eligible articles reporting as TB incidence rate among HIV-positive individuals were summarized according to the target age group, study regions, and study setting. We computed the incidence rates, and confidence intervals for studies that only reported the number of TB cases and person-years of follow-up for the different strata in the cohort, using Stata version 14 software.

Meta-analyses (quantitative reviews) were conducted to determine the overall pooled incidence rate of TB among HIV infected individuals across different categories or strata. Heterogeneity was evaluated using the Cochran statistic and the *I*^2^ statistics [24]. The magnitude of statistical heterogeneity between studies was assessed using *I*^2^ statistics and values of 25, 50, and 75% were considered to represent low, medium, and high, respectively. The random-effects model was used for the data identified as heterogeneous during analysis. Meta-analyses and meta-regression were performed using Stata software version 14.1. For the incidence of tuberculosis with data from ≥10 studies, we performed meta-regression analyses to calculate the hazard ratio (HR). Besides, we carried out a leave-one-out sensitivity analysis to evaluate the key studies that exert a major impact on between-study heterogeneity. The small study effect was assessed by a funnel plot and Egger’s regression tests [24–26].

## Results

### Search results

**Fig. 1:**
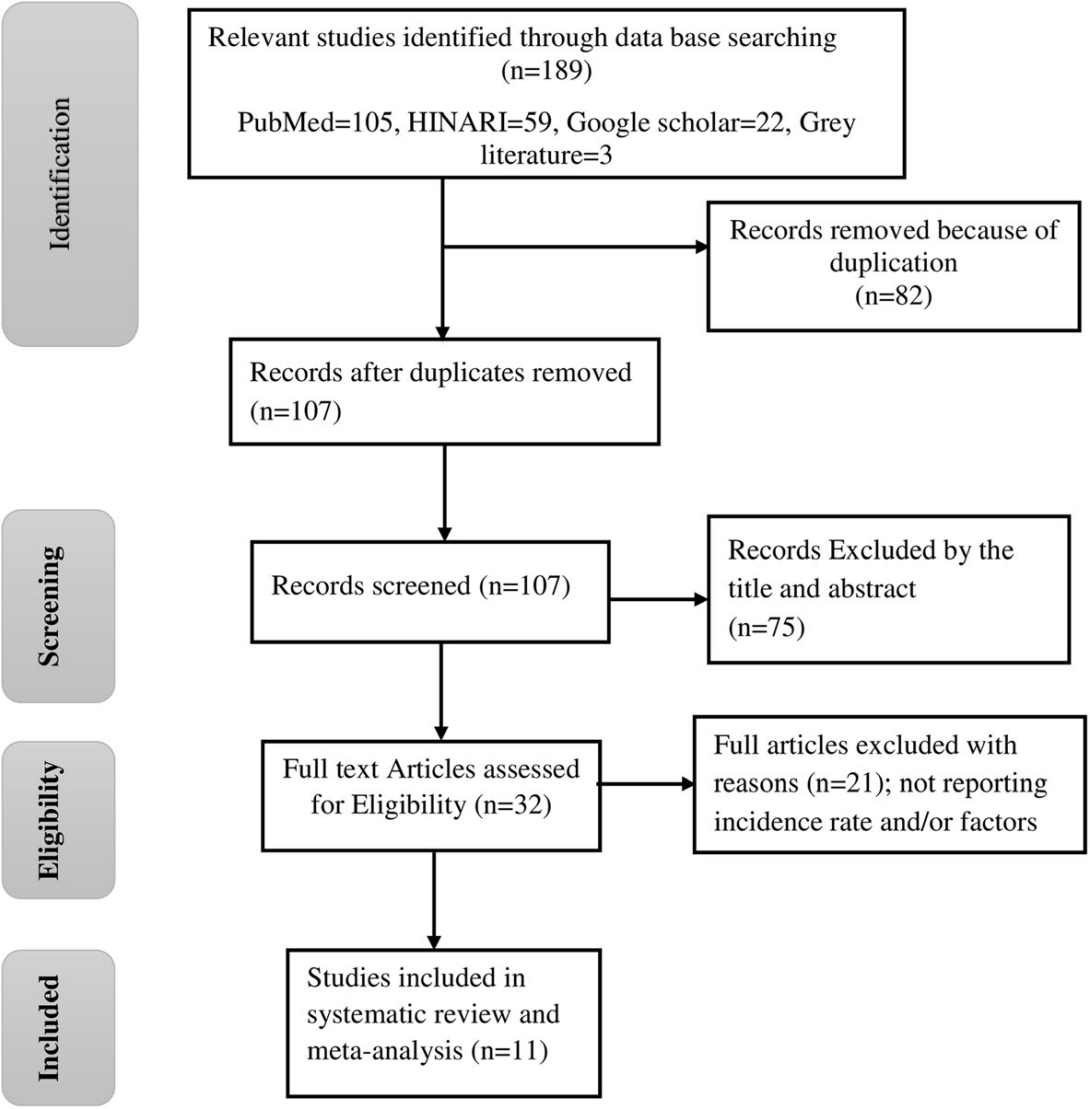
PRISMA flow diagram for the studies screened, reviewed, and included in Ethiopia, 2020

The combined literature search strategy retrieved a total of 189 potential studies, of which 3 records were articles by manual search sources, 32 were screened for full-text review, and 11 studies were eligible to be included in the systematic and meta-analysis (Fig. 1).

### Description and risk bias assessment of the included studies

Eleven articles that were published from 2010 to 2019 in different parts of Ethiopia from international peer-reviewed and national journals included estimating incidence and predictors of tuberculosis among HIV-infected patients. A total sample size of 8875 participants was involved in the final analysis. Among these, 1036 were new tuberculosis cases from 2010 to 2019 published years. The sample size of included studies ranged from 271 [13] to 4210 [22] subjects. All of the reviewed studies were retrospective follow-up studies with a follow-up duration for assessing the outcome ranges from 42 to 84 months [11–15, 17–22].

Findings from a review of studies showed that the highest and lowest incidence rate of tuberculosis among HIV patients was in the Afar [12] and Amhara regions [21] which were 8.64 and 2.22 per 100 person-year observations respectively [12, 21] (Table 1). The majority of the studies reported that being ambulatory and/or bedridden, being anemic, being on WHO clinical staging III and IV, being males, having CD4 less than 200 cells/mm3, having a history of tuberculosis, and being not on CPT and IPT were significant positive predictors for the incidence of tuberculosis among HIV-positive patients after initiation of ART.

### The pooled incidence of TB among HIV-infected patients on ART

**Fig. 2:**
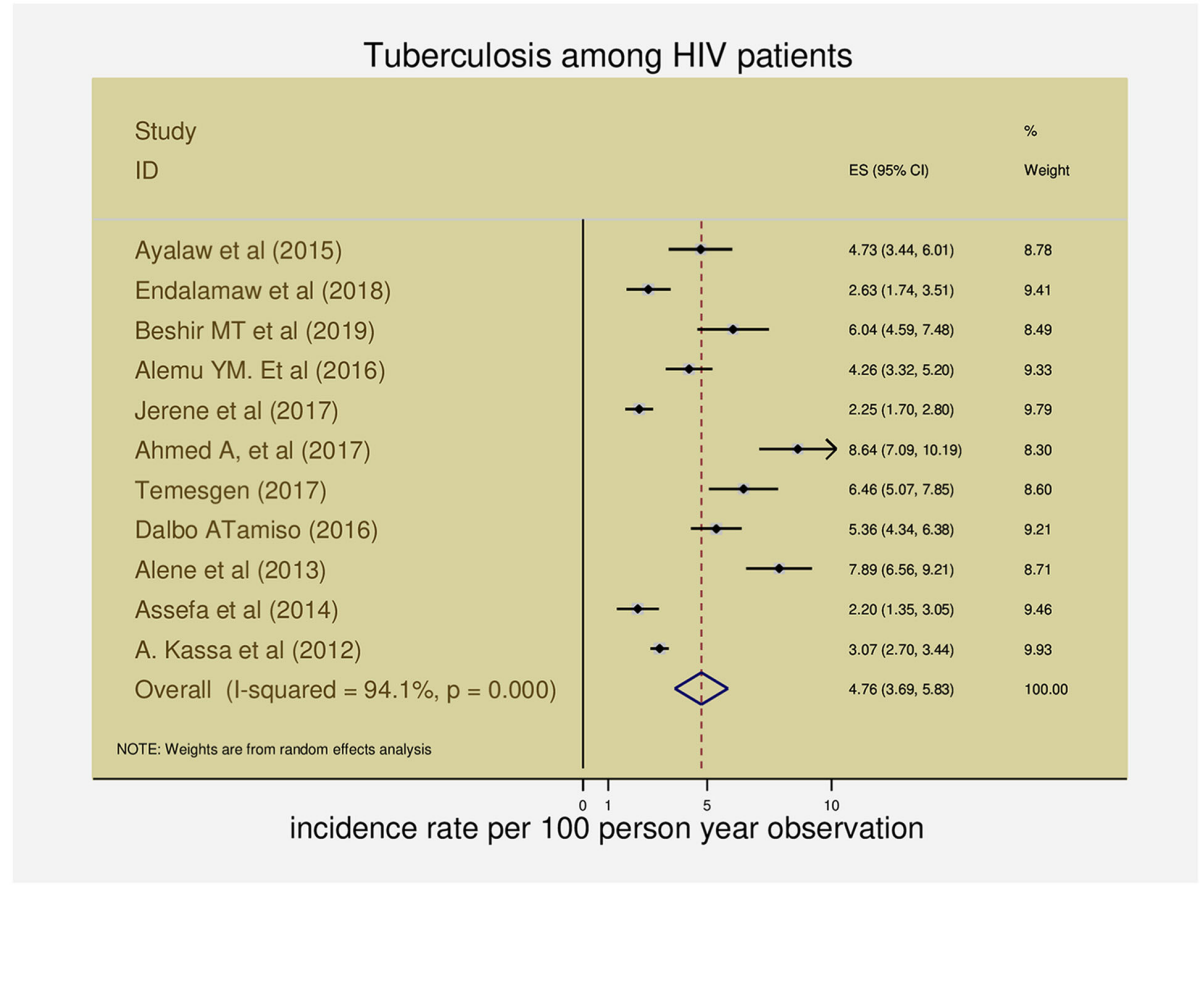
The forest plots of the incidence rate of tuberculosis among HIV-infected patients in Ethiopia

All eleven articles provided information on the incidence of tuberculosis among PLHIV in Ethiopia [11–15, 17–22]. Based on the random-effects method, pooled incidence rate of TB per 100-person-year observations among HIV-infected patients on ART was 4.8 (95% CI 3.69–5.83) and heterogeneity was considerable (*I*^2^ = 94.1%, *Q* = 170, DF = 10, variance= 2.97, *z*=8.7, *p* <0.0001) (Fig. 2).

### Subgroup analyses of tuberculosis incidence among HIV-infected patients on ART

We conducted subgroup analysis based on the pre-defined category of study setting, study population, and length of follow-up time to determine the incidence of tuberculosis.

In our subgroup analysis, the incidence of tuberculosis among PLWHIV with studies in hospital-based was slightly lesser than facility-based studies (4.7; 95% CI 3.46, 5.97 vs 5.0; 95% CI 1.92, 8.01).

We also conducted a subgroup analysis based on the follow-up period for the sampled population. The pooled incidence of tuberculosis among HIV-infected patients after initiation of ART with 60 months or more follow-up period was significantly higher compared to less than 60-month follow-up period (5.1; 95%CI 3.64, 6.46) compared to a longer period of follow-up time (4.1; 95% CI 1.87, 6.27).

In the analyses stratifying summary estimates of TB incidence rates in the above categories, heterogeneity remained high for each stratum. This implied that these variables did not explain most of the heterogeneity observed in the TB incidence rate (Table 2).

**Table 2:** Incidence of tuberculosis in people with HIV in Ethiopia: subgroup meta-analysis and heterogeneity analysis

Subgroup types	Observation (*N*)	IR per 100 PYO	95%CI	*I*^2^ (%)	*Q*-statistic	Tau^2^	Df	*P* value
Study setting								
Hospital based	8	4.7	[3.46,5.97]	93.3	104.9	2.95	7	*P* < 0.0001
Facility based	3	5.0	[1.92,8.01]	96.8	63.4	6.95	2	*P* < 0.0001
Study population								
Children	4	4.3	[2.96,5.71]	83.7	18.5	1.61	3	*P* < 0.0001
Adults	7	5.0	[3.51,6.50]	95.9	148.1	3.77	6	*P* < 0.0001
Follow-up period (months)								
Less than or equal to 60	8	5.1	[3.64,6.46]	94.7	132.8	5.80	7	*P* < 0.0001
Greater than 60	3	4.1	[1.87,6.27]	94.1	34.0	3.53	2	*P* < 0.0001

**Fig. 3:**
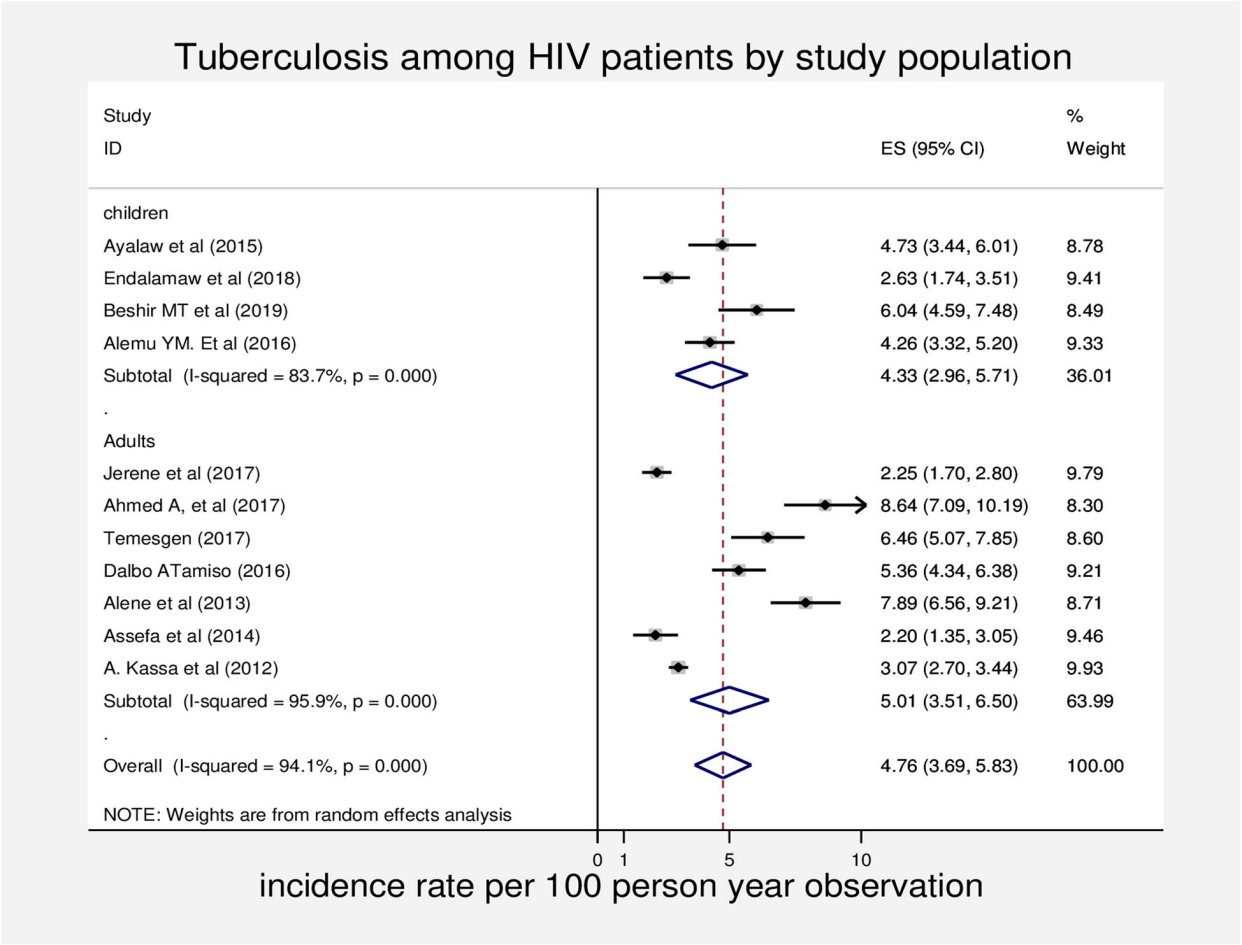
Forest plot of subgroup analysis of the incidence of tuberculosis among HIV-infected patients by study population in Ethiopia

We also performed a subgroup analysis based on the study population to determine the incidence rate of tuberculosis among HIV patients. Four studies were on children and the rest were about adult people. The pooled incidence rate tuberculosis in people living with HIV was 4.3 (95% CI 2.96, 5.71) and 5.0 (95% CI 3.51, 6.50) for the studies conducted on children and adults respectively (Fig. 3).

### Sensitivity analysis

**Fig. 4:**
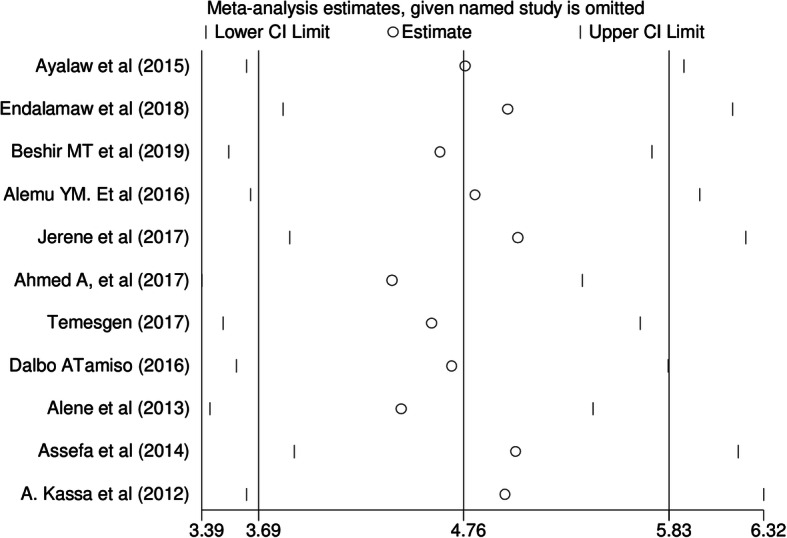
Sensitivity analysis for the incidence of tuberculosis among HIV infected patients in Ethiopia

We performed a leave-one-out sensitivity analysis for the sake of further investigating potential sources of heterogeneity in the analysis of the incidence rate of tuberculosis in PLWHIV. Our sensitivity analysis showed that our findings were not influenced by a single study that all the point estimates of the leave-one-out are within the confidence interval of the combined estimate and it is stable. Our pooled estimated incidence varied between 4.4 (3.39–5.38) and 5.0 (3.85–6.23) after the deletion of a single study (see Table 3 and Fig. 4).

**Table 3:** Sensitivity analysis for the incidence rate of tuberculosis among HIV-infected patients in Ethiopia

Study omitted	Estimate [95% CI]
Ayalaw et al. (2015) [13]	4.8 (3.63, 5.90)
Endalamaw et al. (2018) [17]	5.0 (3.82, 6.16)
Beshir et al. (2019) [15]	4.6 (3.53, 3.74)
Alemu et al. (2016) [18]	4.8 (3.65, 5.99)
Jerene et al. (2017) [19]	5.0 (3.85, 6.23)
Ahmed et al. (2017)	4.4 (3.39, 5.38)
Temesgen et al. (2017)	4.6 (3.51, 5.68)
Dalbo and Tamiso (2016) [20]	4.7 (3.57, 5.82)
Assefa et al. (2014) [21]	5.0 (3.87, 6.19)
Alene et al. (2013) [11]	4.4 (3.43, 5.43)
Kassa et al. (2012) [22]	5.0 (3.63, 6.32)
Combined	4.8 (3.69, 5.83)

### Publication bias

The funnel plot and Egger’s regression tests showed that there is no evidence of substantial publication bias for the incidence of tuberculosis among HIV infected patients in Ethiopia (see Additional files 1 and 2).

### Pooled estimated effects of predictors on the incidence of tuberculosis

Meta-analysis was conducted to identify pooled estimates of predictors for the incidence of tuberculosis among HIV-infected individuals after the initiation of ART in Ethiopia. Among pooled estimates of predictors bedridden functional status, anemia status, WHO clinical staging, cotrimoxazole preventive therapy, isoniazid preventing therapy, CD4 cell count, and gender were found to be significant predictors for the incidence of tuberculosis whereas ambulatory functional status, previous history of tuberculosis, past opportunistic infections, and family size were not statistically significantly pooled predictor estimates for the incidence of tuberculosis among HIV-infected individuals in Ethiopia.

The risk of developing tuberculosis among HIV-infected anemic individuals for the pooled estimates of four observations was 2.3 times more likely as compared to those who had no anemia (HR 2.30; 95%CI 1.75, 3.02; *I*^2^ (*p* value) = 31.2% (0.18); publication bias: *β* (*p* value) = 0.82 (0.60)).

The risk of tuberculosis among HIV-infected individuals who are on WHO clinical stages III and IV was 2.26 times higher than those on WHO clinical stages I and II using meta-analysis of 10 studies (HR 2.26; 95%CI 1.70, 3.02; *I*^2^ (test) = 47.2 % (*p* = 0.048); publication bias: *β* (*p* value) = − 0.13 (0.90)) (Table 4).

**Table 4:** A meta-analysis of predictors of incidence of tuberculosis among HIV-positive patients in Ethiopia (2010–2019)

Variables	Observation	HR	95%CI	*p* value of Q	*I*^2^ (%)	Tau^2^	*Q*-statistic	*P* value of estimate
Functional status								
Working (ref)	4	1						
Ambulatory		1.46	[0.84, 2.51]	*p* = 0.001	78.8	0.29	18.83	*p* = 0.18
Bedridden		2.01	[1.21, 3.35]	*p* = 0.137	42.7%	0.13	6.98	*p* = 0.007
Anemia status								
Not anemic (ref)	8	1						*p* < 0.001
Anemic		2.30	[1.75, 3.02]	*p* = 0.179	31.2	0.05	10.17	
WHO clinical staging								
Stage I/II (ref)	10	1						*p* < 0.001
Stage III/IV		2.26	[1.70, 3.02]	*p* = 0.048	47.2	0.09	17.05	
Cotrimoxazole preventive therapy								
Yes(ref)	5	1						*p* = 0.007
No		2.16	[1.23, 3.72]	*p* = 0.062	55.3	0.20	8.95	
Isoniazid preventing therapy								
Yes (ref)	7	1						*p* = 0.001
No		3.67	[1.73, 7.76]	*p* < 0.001	79.1	0.74	28.7	
CD4 cell count								
Greater than 200	6	1						*p* = 0.002
Less than or equal to 200		2.12	[1.31, 3.43]	*p* < 0.001	90.8	0.32	54.38	
Previous history of tuberculosis								
No	3	1						
Yes		1.49	[0.77, 2.89]	*p* = 0.004	73.5	0.39	15.11	*p* = 0.24
Gender								
Female	5	1						
Male		1.37	[1.16, 1.63]	*p* = 0.588	0	0	2.82	*p* < 0.001
Family size								
Less than five		1						
Five or more	3	1.18	[0.89, 1.56]	*p* = 0.29	19.0	0.01	2.47	*p* = 0.26

The risk of developing tuberculosis among HIV-infected individuals for those not using CPT was 2.16 times more likely than CPT users using five articles (HR 2.16; 95%CI 1.23, 3.72; *I*^2^ (test) = 55.3% (*p* = 0.062); publication bias: *β* (*p* value) = − 1.37 (0.54)).

The risk of TB among male HIV-infected individuals was 37% higher compared to female patients using five articles (HR 1.37; 95%CI 1.16, 1.63; *I*^2^ (test) = 0 (*p* = 0.59); publication bias: *β* (*p* value) = − 2.88 (0.01)).

Since the heterogeneity of the variables not using IPT and CD4 count was high, further analysis by meta-regression was required to manage it (Table 5).

**Table 5:** Meta-regression of selected predictors to the incidence of tuberculosis among HIV-positive patients in Ethiopia (2010–2019)

Study level variables	Low CD4 count				Not using IPT			
	HR with 95% CI	*I*^2^	Tau^2^	Adj. *R*^2^	HR with 95% CI	*I*^2^	Tau^2^	Adj. *R*^2^
Study population								
Children	Ref.							
Adults	2.12 [1.17, 3.86]	90.6%	0.28	0	2.94 [0.50, 17.02]	71.4%	0.52	25.97%
Study setting								
Health facility-based	Ref.							
Hospital based	0.96 [0.21, 4.44]	92.6%	0.36	26.5%	0.31 [0.06, 1.63]	69.2%	0.44	37.93%
Follow-up time (years)								
≤ 5	Ref.							
> 5	2.18 [0.73, 6.50]	78.4%	0.15	46.05%	0.53 [0.06, 5.08]	68.4%	0.68	3.61%

### Meta-regression for high heterogeneity variables

Isoniazid preventive therapy was a statistically significant predictor for the incidence of tuberculosis among HIV infected individuals for seven articles. The risk of developing tuberculosis among HIV-infected individuals for those not using IPT was 3.47 times more likely than IPT users (HR 3.67; 95%CI 1.73,7.76; *I*^2^ (test) = 79.1% (*p* < 0.001); publication bias: *β* (*p* value) = 3.47 (0.14)). Meta-regression revealed that not using IPT in the adults’ study group was higher than children for the incidence of tuberculosis (HR 2.12; 95% CI 1.17, 3.86).

CD4 count at baseline of ART initiation was a significant pooled factor for developing tuberculosis among six articles. The risk of tuberculosis incidence among HIV-infected individuals was 2.12 times more likely for those who had less than 200 cells/mm^3^ at baseline than greater CD4 cell count (HR 2.12; 95%CI 1.31, 3.43; *I*^2^ (test) = 90.8% (*p* < 0.001); publication bias: *β* (*p* value) = − 3.16 (0.57)).

## Discussion

Studies with tuberculosis incidence conducted among HIV patients in different regions of Ethiopia were included and carried out to determine the pooled incidence of tuberculosis among HIV-infected patients after ART initiation. Eleven studies that were published in scientific and reputable journals between 2010 and 2019 were included in this study.

Findings from a systematic review of included studies showed that the highest and lowest incidence rate of tuberculosis among HIV patients was observed in studies conducted in the Afar region and in the Amhara region which was 8.64 and 2.2 per 100 PYO respectively [12].

The current study revealed that the pooled TB incidence among HIV-infected patients after initiation of ART was 4.76 (95% CI 3.69–5.83) per 100 PYO which was in line with the primary studies done in India in 2019 and Tanzania in 2015 which were 4.39 cases and 4.4 cases per 100 PYO, respectively [27, 28]. The current finding of pooled incidence was higher than studies conducted in higher-income countries in 2012, Asia in 2019, Brazil and Nigeria in 2017, and South Africa in 2005 which were 0.3, 0.99, 1.90, 0.57, and 2.44 per 100 person-years, respectively [29–33]. This difference might be because of the progressive development of latent to active TB disease due to the high incidence of HIV compared with higher-income countries [34] and this study also incorporated both children and adults. The other reason for the higher incidence might be a better case-finding strategy in Ethiopia. However, it was lower than the prospective community-based study done in South Africa in 2012 that was 7.4 per 100 PYO [5]. This difference might be all included primary studies in this paper which were retrospective cohort studies and health facility-based studies.

Subgroup analysis by study population of the current study showed that the pooled TB incidence among adults and children was slightly different which were 5.01 (95% CI 3.51, 6.50) and 4.34 (95% CI 2.96, 5.71) respectively. This finding was consistent with studies done in India in 2019 and Tanzania in 2015 which were 4.39 cases and 4.4 cases per 100 PYO, respectively [27, 28]. This might be the same socioeconomic status between these countries.

The current meta-analysis of the predictors showed that being anemic patients were a 2.30 higher risk for incidence of tuberculosis among HIV-infected individuals than non-anemic (HR 2.30, 95% CI 1.75, 3.02). This might be due to the reason that anemia leads to the development of infections including tuberculosis by impairing the function of hemoglobin.

Our findings also showed that patients on advanced WHO clinical stages (III and IV) were 2.26 times greater risk of developing TB (HR 2.26, 95% CI 1.70, 3.02). This was supported by several studies [6, 28, 35]. This is since being stages III and IV will have low CD4 cell count and ultimately be unable to defend against infections including tuberculosis.

This finding also revealed that patients with severe immunosuppression (CD4% <200 cell/mm^3^) were 2.12 times higher risk of developing TB than those with higher CD4 in adults. This was consistent with various studies [28, 29, 32, 35, 36] in which severe immune suppression fastened the progression of latent TB to active disease [2]. This is because HIV kills TB’s protective immune cells among HIV patients. A prospective cohort study done in South Africa showed that TB incidence was nearly 7 times higher risk observed with person-time at CD4 cell counts, 100 cells/mL compared with person-time at CD4 cell counts 700 cells/mL [5].

This meta-analysis study revealed that HIV patients without taking IPT were 3.67 times more at risk of developing new active TB than those taking IPT despite lifelong HAART (HR 3.67, 95% CI 1.73, 7.76). Our study was consistent with the study done in Brazil in 2007 [30] which revealed that TB incidence with no IPT was higher. It was also supported by a meta-analysis study done in Ethiopia in which using IPT reduced TB incidence by 74% [37] and another study done in Ethiopia in 2014 [38]. Several studies also showed that TB incidence among those patients on IPT prophylaxis was reduced compared with none-IPT groups [39].

This study also showed that HIV patients without CPT were 2.16 times more hazards of developing TB than their counterparts [HR 2.2, 95% CI 1.23, 3.72]. This was supported by a study done in Asia in 2019 which revealed CPT reduced TB incidence by 28% [31]. This may be since CPT protects at least five types of opportunistic disease among HIV patients that worsened immunosuppression and progression of the disease.

This study identified that previous TB treatment was not a significant predictor of TB incidence. This was in line with the cohort national study conducted in South Africa [33].

Generally, the current study was focused on the incidence of tuberculosis and its predictors among HIV patients after initiation of ART which is critical to improving the quality of life for HIV-infected patients. As a result, programmers and policymakers in the area of HIV shall take this finding to improve and strengthen the existing strategies especially focusing on HIV patients after ART initiation. Moreover, it shall be taken as a baseline to health workers and other stakeholders to made early screening and prevention of TB in PLHIV as TB can occur in any course of HIV treatment.

## Strengths and limitations of this study

More than one reviewer was involved in this systematic review and meta-analysis, and we used a comprehensive searching strategy. Moreover, during this review, we had also strictly followed the PRISMA guideline. This systematic review and meta-analysis study of TB incidence among HIV infected after ART initiation was the first at national by combining both children and adults.

In this systematic review and meta-analysis, we encountered some limitations. For example, the number of studies that are included is limited, which might affect the pooled estimate of tuberculosis incidence and predictors among HIV-infected individuals in Ethiopia. The retrospective nature of the designs restricts the assessment of all possible predictors that affect the incidence of TB.

## Conclusions

The qualitative part of this review showed that TB incidence ranges from 2.2 to 8.64 per 100-person-year observation (PYO), after initiation of ART whereas the meta-analysis showed the pooled estimate of this TB incidence was higher than in several African and Asian countries. The pooled TB incidence among adults and children was 5.0 and 4.3 per 100 PYO respectively.

This meta-analysis indicated that anemia, WHO clinical staging of three and four, and low CD4 cell counts were of significant effects for the high incidence of TB among HIV-positive patients receiving ART. However, isoniazid preventive therapy helps in reducing the incidence of tuberculosis among HIV-infected patients. Besides, the incidence of tuberculosis has a significant difference between children and adults. Moreover, this study also identified the high incidence of active TB among HIV-infected patients at facility-based areas compared to Hospitals. Thus, the Ethiopian government should strengthen the WHO’s three strategies to reduce the high TB incidence among HIV patients. Besides, clinicians should strengthen ART adherence.

## Supplementary Information

**Additional file 1.** Egger test for publication bias for incidences of tuberculosis in Ethiopia.

**Additional file 2.** The funnel plot and Egger’s regression tests.

## Data Availability

Data will be available from the corresponding author upon request other than the included articles.

## Abbreviations

ART: Antiretroviral treatment
BCG: Bacillus Chalmette–Guerin
CPT: Cotrimoxazole preventive therapy
DM: Diabetes mellitus
HIV: Human immunodeficiency virus
IPT: Isoniazid preventive therapy
OI: Opportunistic infections
PLHIV: People living with human immune virus
PYO: Person-year observation
TB: Tuberculosis
WHO: World Health Organization

## References

[1] World Health Organization. Global tuberculosis report 2018. World Health Organization. 2018. Licence: CC BY-NC-SA 3.0 IGO. https://apps.who.int/iris/handle/10665/274453.

[2] Winter JR, Adamu AL, Gupta RK, Stagg HR, Delpech V, Abubakar I (2018). Tuberculosis infection and disease in people living with HIV in countries with low tuberculosis incidence. Int J Tuberc Lung Dis..

[3] Organisation WH (2019). WHO global tuberculosis report: Advocacy-Toolkit.

[4] Bassett IV, Wang B, Chetty S, Giddy J, Losina E, Mazibuko M (2010). Intensive tuberculosis screening for HIV-infected patients starting antiretroviral therapy in Durban, South Africa. Clin Infect Dis..

[5] Gupta A, Wood R, Kaplan R, Bekker L-G, Lawn SD (2012). Tuberculosis incidence rates during 8 years of follow-up of an antiretroviral treatment cohort in South Africa: comparison with rates in the community. Plos one..

[6] Iliyasu Z, Babashani M. Prevalence and predictors of tuberculosis coinfection among HIV-seropositive patients attending the Aminu Kano Teaching Hospital, northern Nigeria. J Epidemiol. 2009;19(2):81–7. 10.2188/jea.JE20080026 PMC3924118 19265273

[7] Mama M, Manilal A, Tesfa H, Mohammed H, Erbo E (2018). Prevalence of pulmonary tuberculosis and associated factors among HIV positive patients attending antiretroviral therapy clinic at Arba Minch General Hospital, Southern Ethiopia. Open Microbiol J.

[8] Organisation WH (2019). WHO GLOBAL TB REPORT 2019: Country Profiles.

[9] UNAIDS. Joint United Nations Programme on HIV/AIDS (UNAIDS) DATA 2017. Contract No.: UNAIDS/JC2910E. 2017.

[10] Mitku AA, Dessie ZG, Muluneh EK, Workie DL. Prevalence and associated factors of TB/HIV co-infection among HIV Infected patients in Amhara region, Ethiopia. Afr Health Sci. 2016;16(2):588–95. 10.4314/ahs.v16i2.29 PMC4994542 27605976

[11] Addis Alene K, Nega A, Wasie TB (2013). Incidence and predictors of tuberculosis among adult people living with human immunodeficiency virus at the University of Gondar Referral Hospital, Northwest Ethiopia. BMC Infect Dis.

[12] Ahmed A, Mekonnen D, Shiferaw AM, Belayneh F, Yenit MK (2018). Incidence and determinants of tuberculosis infection among adult patients with HIV attending HIV care in north-east Ethiopia: a retrospective cohort study. BMJ Open..

[13] Ayalaw SG, Alene KA, Adane AA (2015). Incidence and predictors of tuberculosis among HIV positive children at the University of Gondar Referral Hospital, Northwest Ethiopia: a retrospective follow-up study. Int Sch Res Notices.

[14] Belisty Temesgen, Getiye Dejenu Kibret, Mekonnen N, Alamirew, and, Alebel A. Incidence and predictors of tuberculosis among HIV positive adults on antiretroviral therapy at Debre Markos Referral Hospital, Northwest Ethiopia: a retrospective record review. 2018. 10.1186/s12889-019-7912-9 PMC6880633 31771552

[15] Beshir MT, Beyene AH, Tlaye KG, Demelew TM (2019). Incidence and predictors of tuberculosis among HIV-positive children at Adama Referral Hospital and Medical College, Oromia, Ethiopia: a retrospective follow-up study. Epidemiol Health.

[16] LA Moher D, Tetzlaff J, Altman DG (2009). Systematic reviews and Meta-analyses. Plos Med..

[17] Endalamaw A, Engeda EH, Tezera N. Incidence of tuberculosis in children on antiretroviral therapy: a retrospective cohort study. BMC Res Notes. 2018;11(1):745–9 10.1186/s13104-018-3846-z PMC6195951 30342550

[18] Alemu YM, Andargie G, Gebeye E (2016). High incidence of tuberculosis in the absence of isoniazid and cotrimoxazole preventive therapy in children living with HIV in Northern Ethiopia: a retrospective follow-up study. Plos One..

[19] Jerene D, Abebe W, Taye K, Suarez PG, Feleke Y, Hallstrom I (2017). Tuberculosis along the continuum of HIV care in a cohort of adolescents living with HIV in Ethiopia. Int J Tuberc Lung Dis..

[20] Dalbo M, Tamiso A (2016). Incidence and predictors of tuberculosis among HIV/AIDS infected patients: a five-year retrospective follow-up study. Adv Infect Dis..

[21] Assefa A, Gelaw B, Getnet G, Yitayew G (2014). The effect of incident tuberculosis on immunological response of HIV patients on highly active antiretroviral therapy at the University of Gondar hospital, northwest Ethiopia: a retrospective follow-up study. BMC Infect Dis.

[22] Kassaa A, Tekab A, Shewaamarec A, Jerened D (2012). Incidence of tuberculosis and early mortality in a large cohort of HIV-infected patients receiving antiretroviral therapy in a tertiary hospital in Addis Ababa, Ethiopia. Transact R Soc Trop Med Hyg.

[23] JBI_Critical_Appraisal-Checklist_for_Cohort_Studies. 2017.

[24] Julian P T Higgins, Simon G Thompson, Deeks JJ, and, Altman DG. Measuring inconsistency in meta-analyses. BMJ. 2009;327:557-60. 10.1136/bmj.327.7414.557 PMC192859 12958120

[25] AL. MBE. A basic introduction to fixed-effect and random-effects models for meta-analysis. Res Syn Meth. 2010(1):97--111. 10.1002/jrsm.12 26061376

[26] Bradburn MJ, Deeks JJ, Altman DG, Sterne J (1999). Meta-analysis in Stata. metan, metacum, and metap.

[27] Gupte AN, Kadam D, Sangle S, Rewari BB, Salvi S, Chavan A (2019). Incidence of tuberculosis in HIV-infected adults on first- and second-line antiretroviral therapy in India. BMC Infect Dis..

[28] Liu E, Makubi A, Drain P, Spiegelman D, Sando D, Li N (2015). Tuberculosis incidence rate and risk factors among HIV-infected adults with access to antiretroviral therapy. AIDS.

[29] Pathmanathan I, Dokubo EK, Shiraishi RW, Agolory SG, Auld AF, Onotu D (2017). Incidence and predictors of tuberculosis among HIV-infected adults after initiation of antiretroviral therapy in Nigeria, 2004-2012. Plos one..

[30] Golub JE, Saraceni V, Cavalcante SC, Pacheco AG, Moulton LH, King BS (2007). The impact of antiretroviral therapy and isoniazid preventive therapy on tuberculosis incidence in HIV-infected patients in Rio de Janeiro, Brazil. AIDS.

[31] Ku SW, Jiamsakul A, Joshi K, Pasayan MKU, Widhani A, Chaiwarith R, et al. Cotrimoxazole prophylaxis decreases tuberculosis risk among Asian patients with HIV. Int AIDS Soc. 2019;22(3):e25264. 10.1002/jia2.25264 PMC6439318 30924281

[32] Collaboration TH-C (2012). Impact of antiretroviral therapy on tuberculosis incidence among HIV-positive patients in high-income countries. Clin Infect Dis..

[33] Lawn SD, Badri M, Wood R (2005). Tuberculosis among HIV-infected patients receiving HAART: long-term incidence and risk factors in a South African cohort. AIDS.

[34] Williams BG, Granich R, De Cock KM, Glaziou P, Sharma A, Dye C (2010). Antiretroviral therapy for tuberculosis control in nine African countries. Proc National Acad Sci..

[35] Auld AF, Mbofana F, Shiraishi RW, Alfredo C, Sanchez M, Ellerbrock TV (2013). Incidence and determinants of tuberculosis among adults initiating antiretroviral therapy–Mozambique, 2004–2008. Plos one..

[36] Suthar AB, Lawn SD, del Amo J, Getahun H, Dye C, Sculier D (2012). Antiretroviral therapy for prevention of tuberculosis in adults with HIV: a systematic review and meta-analysis. Plos Med..

[37] Geremew D, Endalamaw A, Negash M, Eshetie S, Tessema B (2019). The protective effect of isoniazid preventive therapy on tuberculosis incidence among HIV positive patients receiving ART in Ethiopian settings: a meta-analysis. BMC Infect Dis.

[38] Yirdaw KD, Jerene D, Gashu Z, Edginton ME, Kumar AM, Letamo Y (2014). Beneficial effect of isoniazid preventive therapy and antiretroviral therapy on the incidence of tuberculosis in people living with HIV in Ethiopia. Plos One..

[39] Umeokonkwo CD, Segun B, Nguku P, Shakir M, Balogun PN, Bulage L, et al. Effectiveness of isoniazid preventive treatment among patients on antiretroviral treatment in Southeast Nigeria: A retrospective cohort study. J Interv Epidemiol Public Health. 2018;1:9.

