# Supplementary material for: Incidence and predictors of tuberculosis among HIV patients after initiation of antiretroviral treatment in Ethiopia: a systematic review and meta-analysis
Source: Trop Med Health. 2021 Feb 25;49:18. doi: 10.1186/s41182-021-00306-2 (PMC7905193; doi:10.1186/s41182-021-00306-2)
Supplement: Supplementary file 1 — Additional file 1. Egger test for publication bias for incidences of tuberculosis in Ethiopia. [file 41182_2021_306_MOESM1_ESM.docx]

Number of studies = 11 Root MSE=1.45

| Std _Eff | Coeff. | Std. Err. | T | P>\|t\| | [95% Conf. Interval] |
| --- | --- | --- | --- | --- | --- |
| slope | 1.59 | 0.48 | 3.27 | 0.01 | (0.49, 2.68) |
| bias | -0.58 | 4.59 | -0.13 | 0.90 | (-10.94,9.80) |
