# Supplementary material for: Incidence and predictors of tuberculosis among HIV patients after initiation of antiretroviral treatment in Ethiopia: a systematic review and meta-analysis
Source: Trop Med Health. 2021 Feb 25;49:18. doi: 10.1186/s41182-021-00306-2 (PMC7905193; doi:10.1186/s41182-021-00306-2)
Supplement: Supplementary file 2 [file 41182_2021_306_MOESM2_ESM.docx]

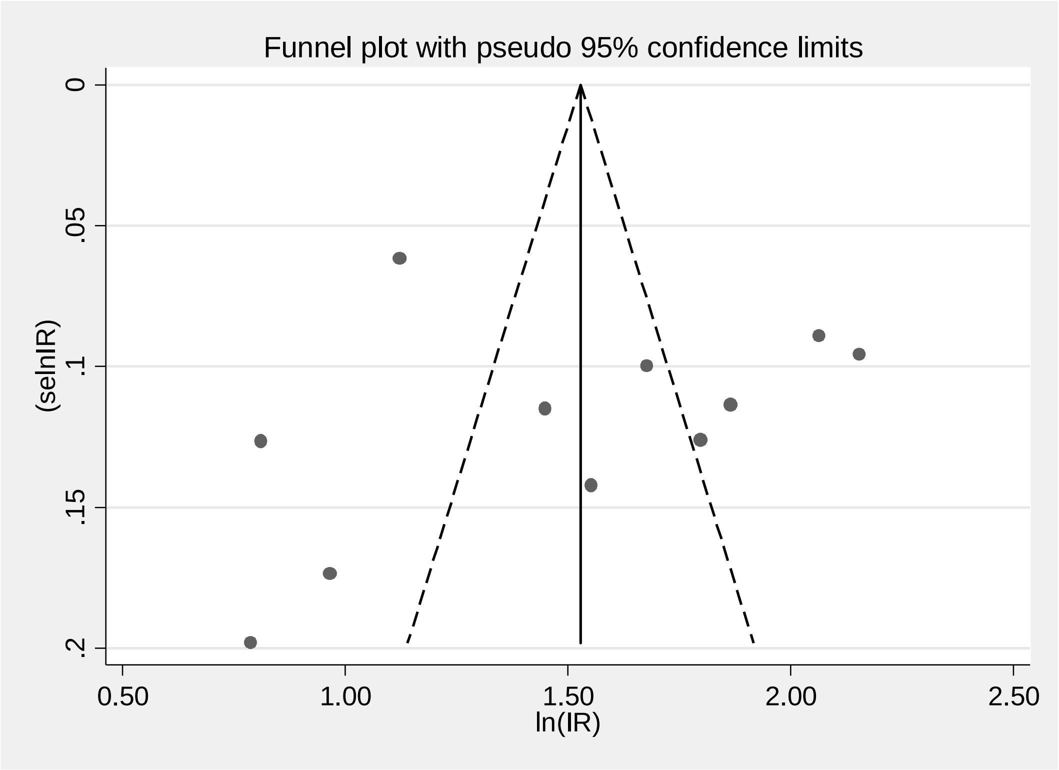

Funnel plot with pseudo 95% confidence limits
0
.05
.1
.15
.2
(selnIR)
0.50
1.00
1.50
2.00
2.50
ln(IR)
